# A combination of routine blood analytes predicts fitness decrement in elderly endurance athletes

**DOI:** 10.1371/journal.pone.0177174

**Published:** 2017-05-05

**Authors:** Helmuth Haslacher, Franz Ratzinger, Thomas Perkmann, Delgerdalai Batmyagmar, Sonja Nistler, Thomas M. Scherzer, Elisabeth Ponocny-Seliger, Alexander Pilger, Marlene Gerner, Vanessa Scheichenberger, Michael Kundi, Georg Endler, Oswald F. Wagner, Robert Winker

**Affiliations:** 1 Department of Laboratory Medicine, Medical University of Vienna, Vienna, Austria; 2 Department of Public Health, Medical University of Vienna, Vienna, Austria; 3 Health and Prevention Center, Sanatorium Hera, Vienna, Austria; 4 Empirical Research, Vienna, Austria; 5 Institute of Occupational Medicine, Medical University of Vienna, Vienna, Austria; 6 Gruppenpraxis Labors.at, Vienna, Austria; Nanyang Technological University, SINGAPORE

## Abstract

Endurance sports are enjoying greater popularity, particularly among new target groups such as the elderly. Predictors of future physical capacities providing a basis for training adaptations are in high demand. We therefore aimed to estimate the future physical performance of elderly marathoners (runners/bicyclists) using a set of easily accessible standard laboratory parameters. To this end, 47 elderly marathon athletes underwent physical examinations including bicycle ergometry and a blood draw at baseline and after a three-year follow-up period. In order to compile a statistical model containing baseline laboratory results allowing prediction of follow-up ergometry performance, the cohort was subgrouped into a model training (n = 25) and a test sample (n = 22). The model containing significant predictors in univariate analysis (alanine aminotransferase, urea, folic acid, myeloperoxidase and total cholesterol) presented with high statistical significance and excellent goodness of fit (R^2^ = 0.789, ROC-AUC = 0.951±0.050) in the model training sample and was validated in the test sample (ROC-AUC = 0.786±0.098). Our results suggest that standard laboratory parameters could be particularly useful for predicting future physical capacity in elderly marathoners. It hence merits further research whether these conclusions can be translated to other disciplines or age groups.

## Introduction

Endurance sports, including marathon running and bicycling, are becoming increasingly popular,[1] in particular among the elderly. Especially distance runners are said to aspire to most efficient training in order to improve their competitiveness.[2] Whereas for elite athletes training intensifications might not necessarily lead to performance increases,[3, 4] the steadily decreasing running times within the cohort of elderly amateur athletes [5] suggest that for this group there is still room for further improvement. Thus feasible models predicting the future development of physical performance with a view to adjusting training strategies are highly warranted.

Most studies aiming to predict the physical performance of endurance athletes concentrated solely on physiological and anthropometric surrogates or on training parameters.[6] In this regard, more than twenty years ago, Williams et al. found a significant link between VO_2 max_ and the time taken to complete a half marathon (r = -0.81, p<0.01).[7] Heil et al. reported highly significant correlations between ergometry parameters and bicycle uphill-times (r = 0.92–0.97).[8] Knechtle et al. predicted running performances of female half-marathoners using anthropometric and training variables such as body weight, skin-fold thickness and mean speed during training sessions.[9]

However, data on the predictive capabilities of blood test results regarding physical performance are still sparse, although routine blood tests could be easily performed and blood markers might provide a valuable contribution to prediction models, since it is clearly established that recurrent exercise alters a high number of biochemical markers.[10–12] For instance, the effect of exercise on blood lipid composition is well established.[13] Less well known are the results of two independent groups [14, 15] describing low pre-run folate levels in marathon runners, one of these studies concluding that folate substitution had no effect on treadmill performance [14]. Moreover, Suzuki et al., Henrotin et al. and Hessel et al. reported higher levels of myeloperoxidase [16, 17] or lipid peroxides [18] during or after endurance running.

However, scientific results, especially laboratory tests, still have only a small effect on training adaptions, which is thought to be due to the fact that current training methods primarily developed by trial and error, since scientific evidence is obviously too limited to be considered.[4] Among the few relevant studies, Lippi et al. quantified a set of standard laboratory parameters shortly before a half-marathon run and found a significant association between increased blood platelet volumes and running performance.[6] In another report, the same group identified α-amylase, creatine kinase, blood glucose, high-density lipoprotein-cholesteron, lactate dehydrogenase, urea and uric acid as univariate predictors of half-marathon running performance when studying 43 amateur runners. Among those markers, α-amylase remained statistically significant after multivariate analysis.[19] Bobbert and coworkers reported correlations between marathon times and recent levels of circulating hormones, and especially for leptin (r = 0.607, p < .001).[20]

Nevertheless, there is still an absence of longitudinal studies particularly assessing the predictability of medium- to long-term performance changes. We thus aimed to predict performance drops at a later point in time by routine blood parameters in a cohort of elderly marathon runners and bicyclists. Accordingly, our primary hypothesis was that the probability of these performance drops would depend on a set of baseline laboratory results (identified by univariate analysis). The probability could hence be calculated from these specific blood markers by means of binary logistic regression. The random division of our study cohort into a model training sample and a test (or validation) sample subsequently allows for validation of the computed model parameters in an independent sample.

## Materials and methods

### Sample

The study population of this prospective observational study comprises 47 elderly marathon runners and endurance bicyclists (♀ = 4) who–out of a total of 63 screened athletes–met the applied inclusion criteria at baseline and could be re-evaluated after a three-year follow-up period. The reporting of the study conforms to the STROBE statement.[21] Further details on the design of the underlying Vienna Marathon trial can be derived from previous articles.[22–26]

We included male and female individuals aged ≥60 years who had participated in at least one local competition during the previous three years (Wachau Half Marathon, Vienna City Marathon, Carinthian Marathon) and who reported a present training volume of ≥2 hours per week. In contrast, clinically manifest cardiovascular diseases, chronic alcoholism (>60g daily intake or diagnosed history of alcohol abusus) or unwillingness to give written informed consent led to study exclusion.

The Austrian Marathon study (APSOEM) was evaluated and approved by the local ethics committee of the Medical University of Vienna (assigned reference number: EK 401/2005) and registered with ClinicalTrials.gov (NCT01045031). All medical procedures conformed to institutional guidelines on good scientific practice as well as to the Declaration of Helsinki and its further amendments. Each participant gave written informed consent prior to study inclusion.

### Biochemical analytes

Venous blood was drawn at baseline (approx. three years before performance reevaluation) between 10:00 and 10:30 a.m. in order to account for circadian variation. Following an explorative approach, a broad variety of biochemical analytes was chosen as a starting point for univariable correlation analysis. The available set of blood tests has been elaborated after weighing costs (costs for laboratory tests) and possible benefits (higher model accuracy due to a larger number of possible predictors) and was chosen in a way that each parameter represents one or more physiological systems or pathological conditions, as presented in Table 1. Baseline laboratory parameters were assessed at Labors.at, a Viennese Institute for medical laboratory diagnostics (hemoglobin *Hb*, total cholesterol *Chol*, low density lipoprotein/high density lipoprotein ratio *LDL/HDL*, triglycerides *Trig*, aspartate aminotransferase ASAT, alanine aminotransferase ALAT, blood urea nitrogen *BUN*, creatinine *Crea*, folic acid *Fol*), or at the Department of Laboratory Medicine, Medical University of Vienna (25(OH) vitamin D *25(OH)D*, insulin-like growth factor 1 *IGF-1*, myeloperoxidase *MPO*) in a certified (ISO 9001) and accredited (ISO 15189) environment. Furthermore, the last three compounds were quantified from frozen sera which until then were stored at the MedUni Wien Biobank facility (www.biobank.at). Detailed information about quantification methods can be derived from Table 1.

**Table 1:** Detection methods for biochemical parameters.

**Analyte**	**Method**	**Assay/Platform**	**Reason why selected**
**Hb**	*Photometric*	*ADVIA 2120i Hematology System (Siemens Healthcare Diagnostics*, *Eschborn*, *Germany)*	Oxygen capacity [27, 28]
**Chol**	*Enzymatic colorimetry (CHOD-PAP)*	*Abbott Architect c8000 Clinical Chemistry Analyzer (Abbott Laboratories*, *Abbott Park*, *USA)*	Tendon structure [29], Lipid lowering capacity of exercise [30, 31]
**LDL/HDL**	*Inhibition method*	*Abbott Architect c8000 Clinical Chemistry Analyzer (Abbott Laboratories)*	
**Trig**	*Enzymatic colorimetry (GPO-PAP)*	*Abbott Architect c8000 Clinical Chemistry Analyzer (Abbott Laboratories)*	
**ASAT**	*IFCC method*	*Abbott Architect c8000 Clinical Chemistry Analyzer (Abbott Laboratories)*	Liver metabolism [32]
**ALAT**	*IFCC method*	*Abbott Architect c8000 Clinical Chemistry Analyzer (Abbott Laboratories)*	
**BUN**	*Urease-GLDH method*	*Abbott Architect c8000 Clinical Chemistry Analyzer (Abbott Laboratories)*	Liver metabolism, Hemolysis [32]
**Crea**	*Jaffe method*	*Abbott Architect c8000 Clinical Chemistry Analyzer (Abbott Laboratories)*	Kidney function [32], Muscle mass [32]
**Folic acid**	*CLIA*	*Abbott Architect c8000 Clinical Chemistry Analyzer (Abbott Laboratories)*	Increased consumption during exercise [15]
**21(OH)D**	*CLIA*	*LIAISON® 25 OH Vitamin D TOTAL Assay*, *LIAISON® analyzer (DiaSorin S*.*p*.*A*., *Saluggia*, *Italy)*	Osteoporosis [33], Athletic performance [34]
**MPO**	*ELISA*	*Quantikine ® Human MPO Immunoassay kit (R&D Systems*, *Inc*., *Minneapolis*, *USA)*	Inflammation, Oxidative Stress [16, 17, 35]
**IGF-1**	*CLIA*	*LIAISON® IGF-I*, *LIAISON® analyzer (DiaSorin S*.*p*.*A*.*)*	HPG axis, body fat [36]

*Hb … hemoglobin*, *Chol … total cholesterol*, *HDL/LDL … ratio of high densitiy lipoprotein to low-density lipoprotein*, *Trig … triglycerides*, *ASAT … aspartate aminotransferase*, *ALAT … alanine aminotransferase*, *BUN … blood urea nitrogen*, *Crea … Creatinine*, *21(OH)D … 21(OH) Vitamin D*_*3*_, *MPO … myeloperoxidase*, *IGF-1 insulin-like growth factor 1*, *ELISA … enzyme-linked immunosorbent assay*, *CLIA … chemiluminescence immunoassay*, *CHOD … cholesterol oxidase*, *GOD … glucose oxidase*, *PAP … phenol + aminophenazone*, *IFCC … International Federation of Clinical Chemistry and Laboratory Medicine*, *GLDH … glutamate dehydrogenase*, *HPG …* hypothalamic–pituitary–gonadal

### Physical performance

Physical performance was estimated by means of ergometry (Ergometrics 900, ergoline GmbH, Bitz, Germany) after at least one rest day before the examination day. Starting with 25W, workload was increased every two minutes by 25W until the point of exhaustion was reached. The maximum workload given in [W] reached at the point of exhaustion represents the individual absolute physical performance in [W]. Relative physical performance was then calculated as the individualmaximum workload achieved calculated as a percentage of the final wattage relative to tabulated sex, age and body surface-specific performance references.[37]

The participants’ individual differences in physical performance between baseline and follow up were calculated as

$${relative\;physical\;performance}_{follow-up}-relative\;{phyiscal\;performance}_{baseline}.$$

Accordingly, a performance drop between baseline and follow-up is defined as any negative result of this expression.

### Statistical analyses

Continuous data is given as median (interquartile range), or, if indicated, as median and bootstrapped 95% confidence interval of the median. Categorical data is presented as counts and percentages. Correlation coefficients between metric variables are expressed as Spearman’s ρ. Possible differences of continuous data between both samples were compared by Mann-Whitney U-Tests and Wilcoxon tests (paired data) respectively.

Performance drops between baseline and follow-up were predicted by means of binary logistic regression models. For this, the total sample was randomly divided (random generator, SPSS 23, IBM, Armonk, USA) into a model training (N = 25) and a test (N = 22) sample. The idea behind this statistical approach is to validate a model, which is compiled using only data of the model training sample, within the test sample. A prediction model that was calculated solely within the test sample, would not show any predictive value within the independent test sample, if it was only a result of chance. In contrast, a successful translation of the model to the test sample (significant predictive capabilities in both, the model training and the test sample) would be indicative of a valid model. In detail, variables presenting with a statistically significant univariate correlation with the difference in physical performance between baseline and follow-up where chosen as predictors. Predicted probabilities for a performance drop were estimated within the model training sample and quality criteria of the model were assessed by means of ROC (receiver-operator curve) plots, Youden index and contingency tables. The model parameters derived from the model training sample were then used to generate the model’s regression equation, by which probabilities of a performance drop could also be predicted for the test sample, which was then used to validate the model.

All p-values must be interpreted two-sided.

All calculations and graphical depictions were performed using SPSS 23 (IBM), MedCalc Statistical Software version 15.8 (MedCalc Software bvba, Ostend, Belgium) and SigmaPlot 13 (Systat Software Inc, San Jose, USA).

## Results

47 athletes were enrolled into the present evaluation, four of them women. The median age of the total study population (N = 47, ♀ = 4) was 65 years (Q1–Q3: 61–68). Except for total cholesterol (<200 mg/dl) and 25(OH)D (75–250 nMol), all biochemical parameters were within the reference range. The relative physical performance amounted to 152% (127.9–169.2). There were no statistically significant differences between the model training and the test sample with regards to all assessed parameters. Detailed information can be derived from Table 2.

**Table 2:** Distribution of biochemical and physiological parameters in the total study population, as well as separated by group affiliation (model training sample N = 25/test sample N = 22) at baseline (columns 2–4) and follow-up (columns 6–8), given as median and bootstrapped 95% confidence interval of the median.

	**Total sample**	**Model training sample**	**Test sample**	**p-Value**	**Total sample**	**Model training sample**	**Test sample**	**p-Value**
	*Baseline*				*Follow up*			
**Age [y]**	*65 (63–67)*	*65 (61–70)*	*65 (63–67)*	.*740*	*69 (67–71)*	*69 (65–73)*	*68 (66–71)*	.*572*
**Hemoglobin [g/dl]**	*14*.*3 (14*.*1–14*.*6)*	*14*.*3 (13*.*6–14*.*8)*	*14*.*4 (14*.*1–14*.*9)*	.*550*	*14*.*5 (13*.*9–14*.*6)*	*14*.*5 (13*.*7–14*.*7)*	*14*.*5 (13*.*9–15*.*0)*	.*258*
**Cholesterol [mg/dl]**	*224 (214–232)*	*225 (204–233)*	*223 (201–237)*	.*924*	*228 (210–241)*	*221 (205–232)*	*237 (212–252)*	.*122*
**LDL/HDL Ratio**	*2*.*0 (1*.*8–2*.*5)*	*1*.*9 (1*.*7–2*.*7)*	*2*.*1 (1*.*8–2*.*7)*	.*651*	*2*.*3 (1*.*9–2*.*6)*	*2*.*1 (1*.*7–2*.*7)*	*2*.*3 (1*.*9–2*.*7)*	.*343*
**Triglycerides [mg/dl]**	*127 (108–136)*	*134 (101–146)*	*122 (103–139)*	.*815*	*114 (93–128)*	*102 (92–122)*	*128 (90–149)*	.*254*
**ASAT [U/l]**	*28 (27–30)*	*28 (24–34)*	*28 (27–31)*	.*600*	*28 (27–30)*	*29 (25–32)*	*28 (26–30)*	.*881*
**ALAT [U/L]**	*24 (22–25)*	*23 (22–25)*	*25 (21–29)*	.*474*	*23 (20–26)*	*23 (19–29)*	*23 (20–26)*	.*757*
**BUN [mg/dl]**	*17 (16–19)*	*17 (15–19)*	*18 (16–20)*	.*724*	*19 (17–20)*	*18 (16–20)*	*19 (17–20)*	.*282*
**Creatinine [mg/dl]**	*0*.*9 (0*.*9–1*.*0)*	*1*.*0 (0*.*9–1*.*0)*	*0*.*9 (0*.*9–1*.*0)*	.*678*	*1*.*0 (0*.*9–1*.*0)*	*1*.*0 (0*.*9–1*.*0)*	*1*.*0 (0*.*9–1*.*0)*	.*798*
**Folic acid [ng/ml]**	*10*.*4 (9*.*8–12*.*0)*	*10*.*4 (8*.*2–12*.*0)*	*10*.*9 (9*.*3–12*.*7)*	.*550*	*17*.*1 (14*.*5–18*.*7)*	*16*.*9 (14*.*0–18*.*4)*	*18*.*7 (112*.*8–24*.*6)*	.*543*
**25(OH)D [nMol]**	*54*.*1 (46*.*3–58*.*1)*	*53*.*4 (41*.*9–57*.*3)*	*55*.*6 (40*.*7–63*.*3)*	.*572*	*71*.*8 (67*.*5–81*.*7)*	*67*.*7 (64*.*7–86*.*5)*	*74*.*5 (69*.*6–82*.*1)*	.*522*
**MPO [ng/ml]**	*876*.*0 (740*.*0–1127*.*6)*	*802*.*9 (667*.*0–1292*.*5)*	*889*.*3 (622*.*1–1213*.*3)*	.*798*	*–*	*–*	*–*	*–*
**IGF-1 [ng/ml]**	*137 (114–148)*	*119 (103–154)*	*140 (121–148)*	.*436*	*162 (145–176)*	*152 (133–179)*	*168 (152–180)*	.*232*
**Training volume [h/week]**	*5*.*0 (4*.*5–6*.*0)*	*5*.*5 (4*.*0–8*.*0)*	*5*.*0 (4*.*0–6*.*0)*	.*352*	*6*.*5 (5*.*0–8*.*0)*	*8*.*0 (5*.*0–10*.*0)*	*6*.*0 (3*.*5–8*.*0)*	.*138*
**Absolute physical performance [W]**	*200 (188–225)*	*200 (188–250)*	*194 (175–219)*	.*069*	*200 (177–222)*	*220 (178–225)*	*187 (169–218)*	.*112*
**Relative physical performance [%]**	*152 (144–167)*	*155 (144–169)*	*150 (129–169)*	.*232*	*149 (134–156)*	*153 (134–164)*	*139 (123–159)*	.*208*

MPO was measured only at baseline. The p-values are derived from Mann-Whitney U tests assessing differences between the model training and the test sample.

### Parameter selection

**Fig 1:**
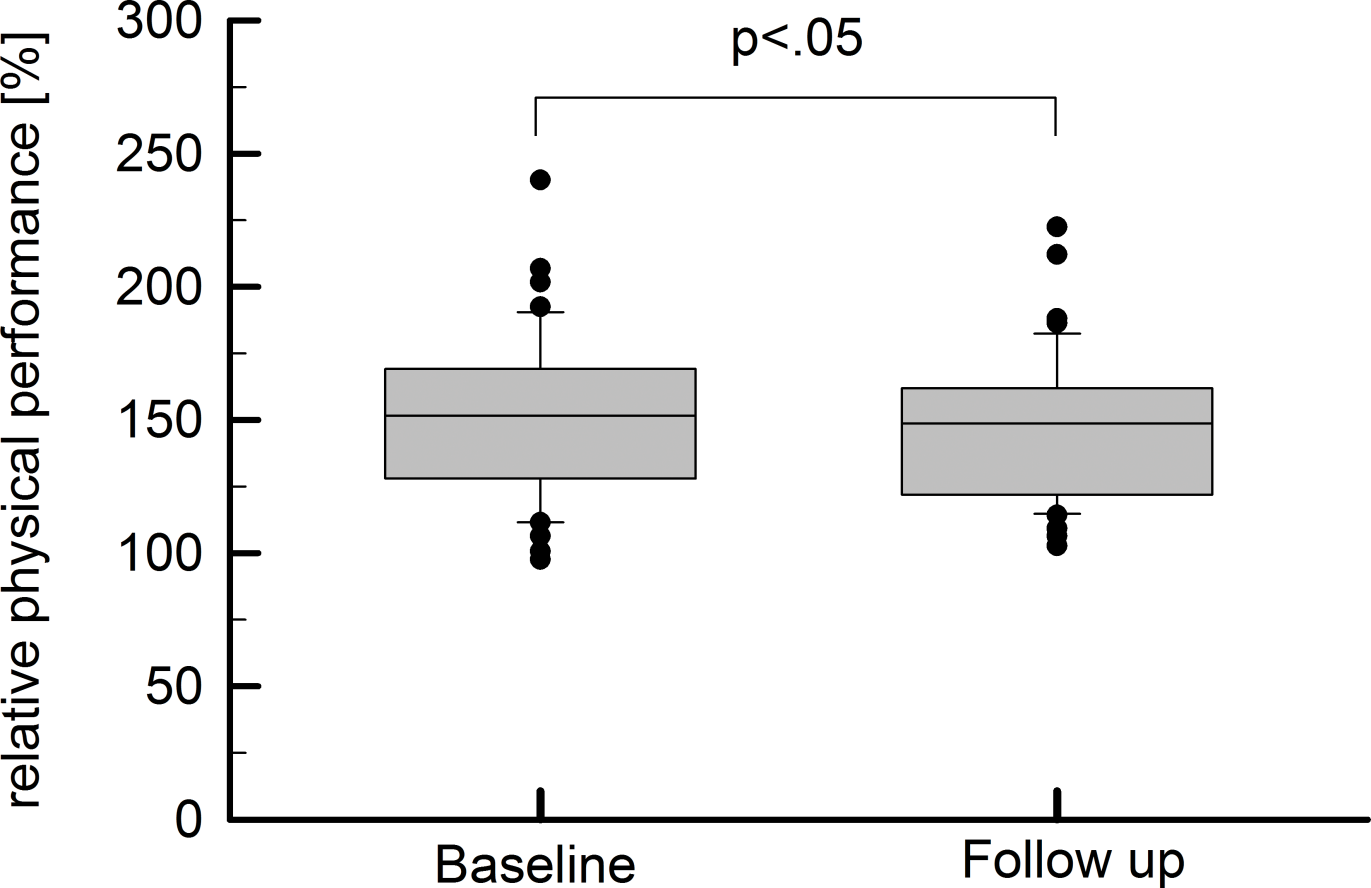
Differences between relative physical performance at baseline and follow-up examinations were assessed by Wilcoxon tests and led to a statistically significant result (p < .05).

Between baseline and follow up, there was a slight decrease in relative physical performance: 152% (128–169) vs. 149% (122–162), Z = -2.036, p = .042 (see Fig 1). In detail, 64% of the total study population experienced a performance drop with a median decrease of 16% (12–22%). The remaining individuals presented with a median improvement of 18% (3–20). For selection of model coefficients, univariate correlations between performance changes and biochemical parameters were undertaken (see Table 3) within the model training sample. The following parameters with significant correlation coefficients were selected as predictor variables for subsequent binary logistic regression models: total cholesterol (ρ = -4.060, p = .021), ALAT (ρ = 0.526, p = .007), BUN (ρ = 0.476, p = .016), folic acid (ρ = -0.521, p = .008) and MPO (ρ = 0.404, p = .045).

**Table 3:** Univariate correlations between differences in ergometry performance and distinct laboratory parameters. Correlation coefficients are given as Spearman’s ρ.

	**Age**	**BMI**	**Hb**	**Chol**	**LDL/HDL**	**Trig**	**ASAT**	**ALAT**	**BUN**
Δ_performance_	*-0*.*244*	*0*.*033*	*-0*.*086*	*-0*.460*	*-0*.*038*	*0*.*322*	*0*.*207*	*0*.526**	*0*.476*
	**Crea**	**Folic acid**	**21(OH)D**	**MPO**	**IGF-1**				
Δ_performance_	*-0*.*143*	*-0*.521**	*-0*.*047*	*0*.404*	*-0*.*294*				

Δ_performance_ … difference in physical performance between baseline and follow up, Hb … hemoglobin, Chol … total cholesterol, HDL/LDL … ratio of high densitiy lipoprotein to low-density lipoprotein, Trig … triglycerides, ASAT … aspartate aminotransferase, ALAT … alanine aminotransferase, BUN … blood urea nitrogen, Crea … Creatinine, 21(OH)D … 21(OH) Vitamin D_3_, MPO … myeloperoxidase, IGF1- insulin like growth factor 1

* p < .05

** p < .01.

### Model design

A binary logistic regression model predicting a performance drop (between baseline and follow up) by baseline total cholesterol, ALAT, BUN, folic acid and MPO levels was compiled within the model training sample:

$$P(Y=1)=\frac{e^{\text{-}21.368+0.070\times \text{chol}+1.315\times \text{folic}\;\mathrm{acid}\text{-}0.478\times \text{ALAT}+0.273\times \text{BUN}+0.0006\times \text{MPO}}}{1+e^{\text{-}21.368+0.070\times \text{chol}+1.315\times \text{folic}\;\text{acid-}0.478\times \text{ALAT}+0.273\times \text{BUN}+0.0006\times \text{MPO}}}$$

**Fig 2:**
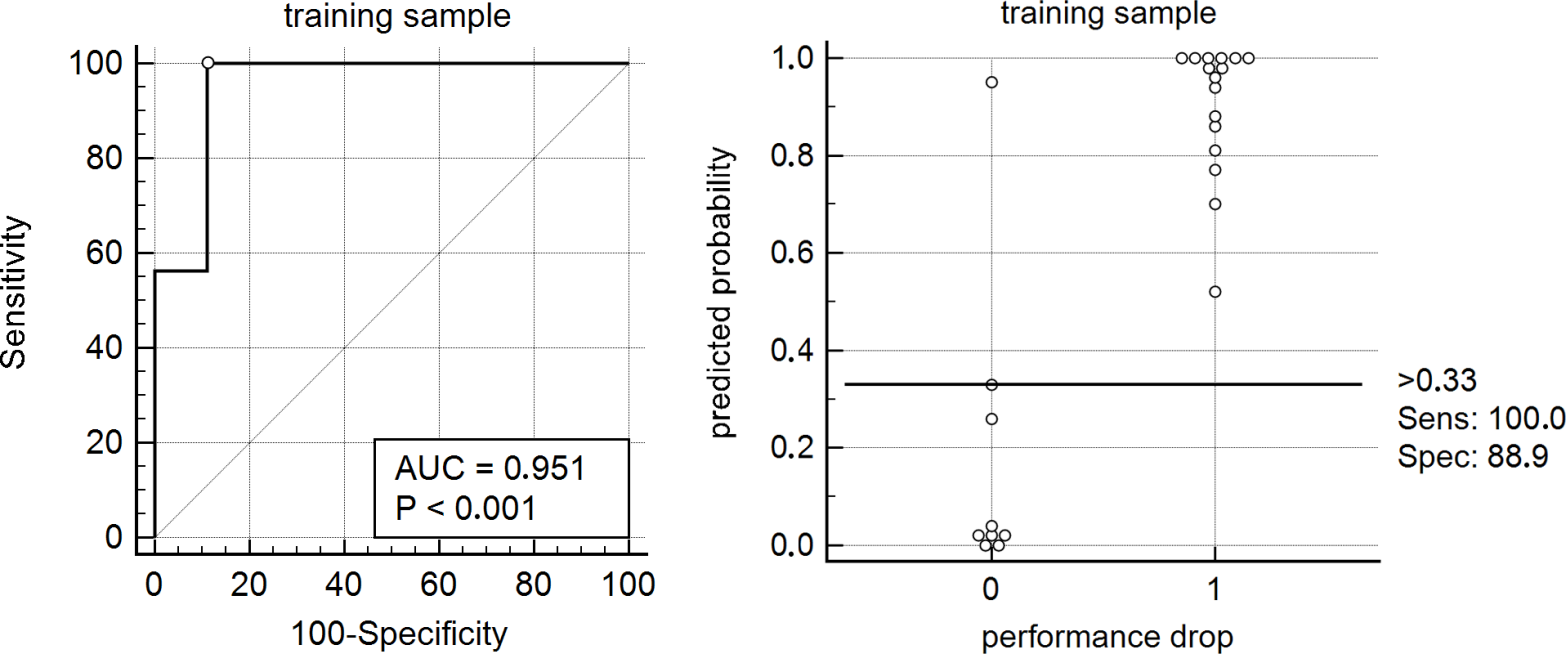
A binary logistic regression model containing the significant univariate predictors of future performance (alanine aminotransferase, urea, folic acid, myeloperoxidase, total cholesterol) with future performance drop as an outcome variable was trained. The resulting model presented with high statistical significance and excellent goodness of fit.

In subsequent ROC-analysis, the highly significant model (χ^2^ = 21.412, df = 5, p = .001) yielded an excellent[38] area under the curve (AUC): 0.951±0.050, p<0.0001 (see Fig 2). According to the Youden index method, an estimated probability of >0.33 was the optimal cut-off for prediction of performance drops, leading to 100% sensitivity and 89% specificity (see Table 4).

**Table 4:** Quality criteria of the model within the model training sample.

**Quality criterion**	**Model training sample**	**Test sample**	**Difference**
**Global significance**	*χ^2^ = 21*.*412*, *df = 5*, *p =* .*001*		
**Nagelkerke’s R^2^**	*0*.*789*		
**ROC-AUC**	*0*.*951*±*0*.*050*, *p<0*.*0001*	*0*.*786*±*0*.*098*, *p = 0*.*0035*	*Z = 1*.*498*, *p = 0*.*134*
**Youden index**	*0*.*8889 at P>0*.*33*		
**Sensitivity**	*100%*	*79%*	
**Specificity**	*89%*	*63%*	
**PPV**	*84%*	*54%*	
**NPV**	*100%*	*84%*	

PPV … positive predictive value, NPV … negative predictive value

### Model validation

**Fig 3:**
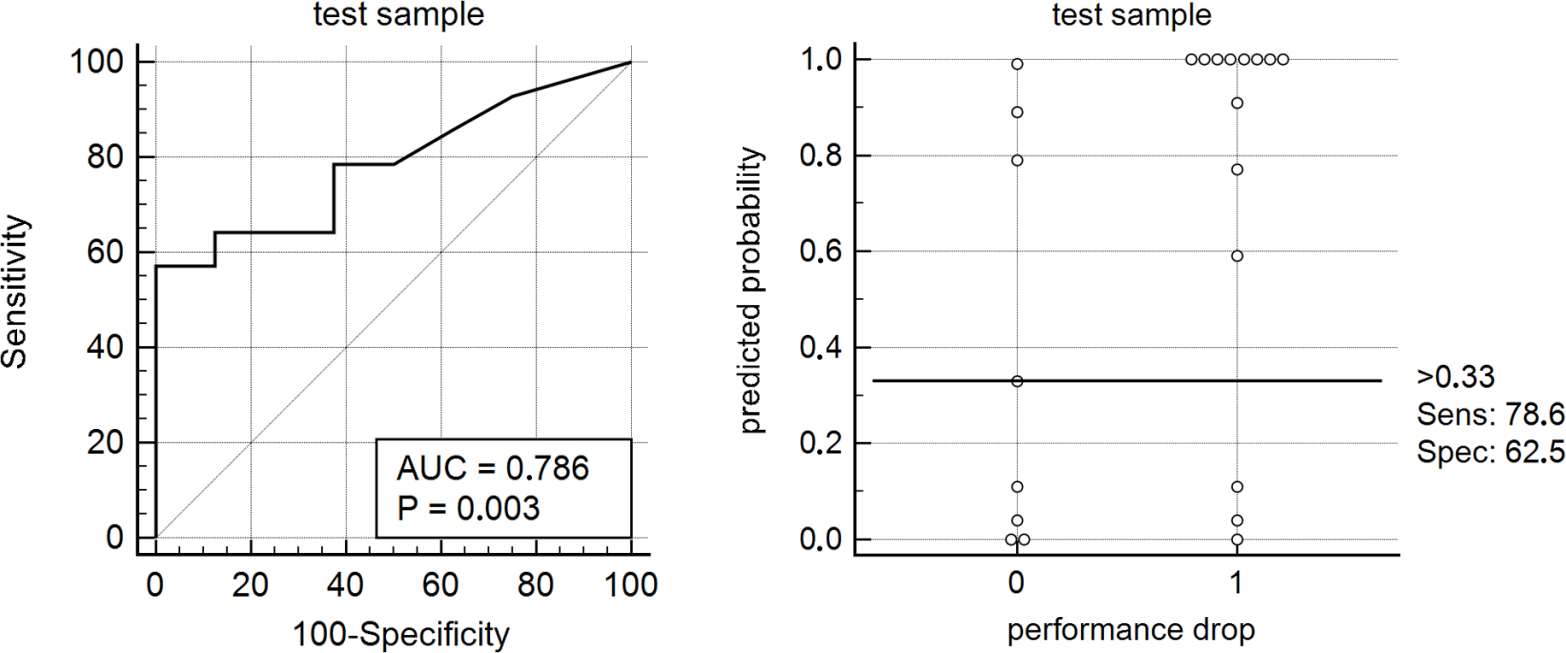
The model parameters were validated in the test sample. The resulting c-statistics (AUC = 0.786) did not significantly differ from the training models’ AUC (p = 0.134).

As described above, the probabilities for performance drops were predicted for individuals in the test sample using the model parameters of the model training sample. The area under the resulting ROC-curve was not significantly different from that of the model training sample: 0.786±0.098, Z = 1.498, p = 0.134. When applying the identical cut-off (<0.33), a decrease in performance was found with 79% sensitivity and 63% specificity (see Table 3, Fig 3).

**Fig 4:**
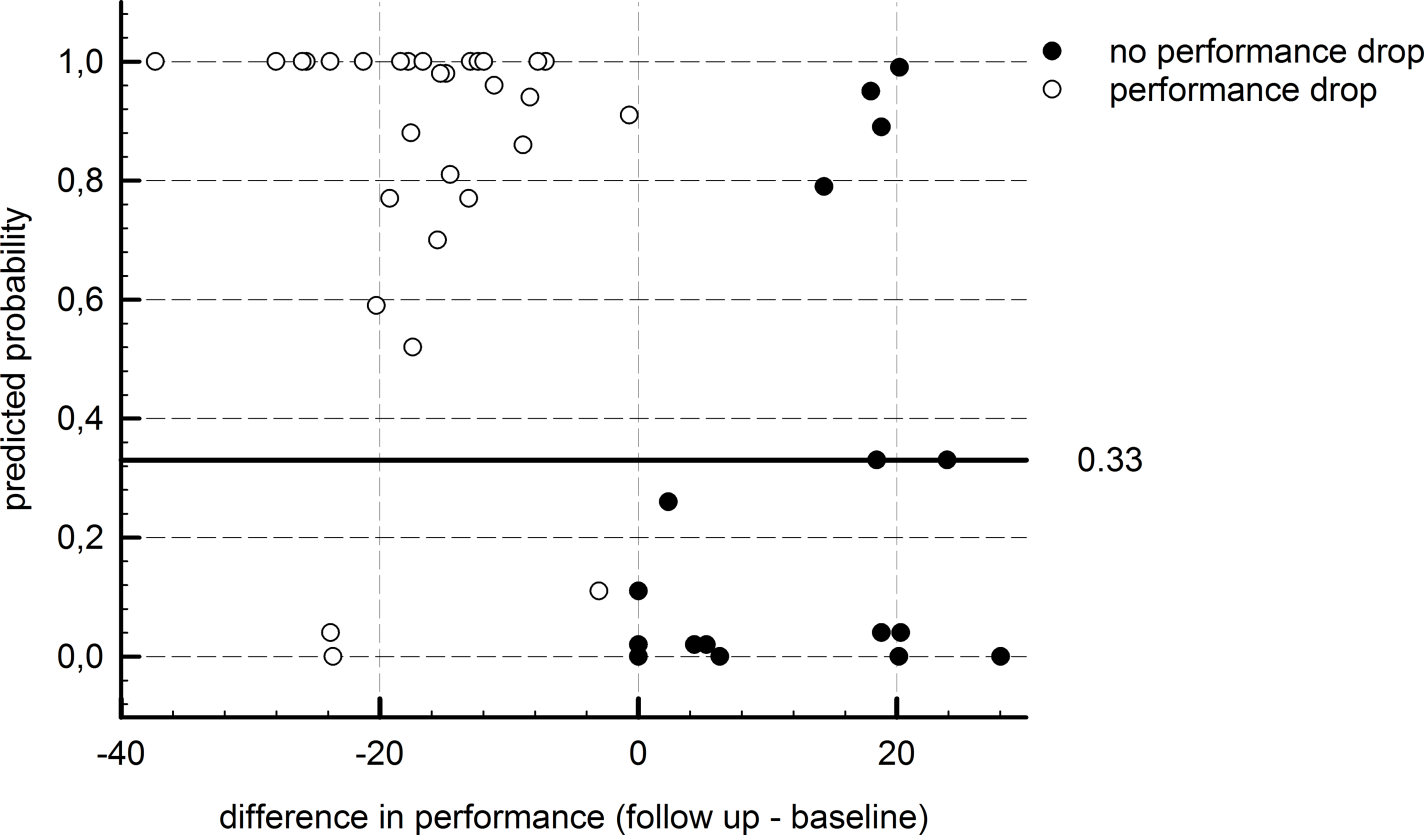
Plot of predicted versus observed data including the whole study population.

Also, for the total study population (training + test sample), the ROC-analysis resulted in highly significant F-statistics (AUC = 0.868, p < .001). Predicted versus observed data is depicted in Fig 4.

## Discussion

Valid predictions of future physical performance creating a possibility to respond to an imminent fitness decrement are in high demand.[4, 6] Our proposed model presented with high statistical significance (χ^2^ = 21.412, df = 5, p = .001) as well as with excellent predictive quality within the model training sample (ROC AUC = 0.951±0.050) and fair c-statistics in the test sample (ROC AUC = 0.786±0.098). The model contained five biochemical parameters, which were identified as significant predictors in an explorative univariate correlation analysis: total cholesterol, blood urea nitrogen, alanine aminotransferase, folic acid and myeloperoxidase. All selected biochemical analytes can be seen as indicators of persistent training activity.

### Mechanistic considerations

#### Muscle breakdown parameters and liver injury

Intensive physical exercise leads to muscle breakdown,[39] and as a consequence to the release of muscle-born enzymes and degradation products such as transaminases and urea.[40] However, especially the increment in ALAT, which is produced by the liver as well, might be a result of exercise-induced liver damage. Shin and coworkers found that the changes in hepatic metabolism depended on the running distance.[41] From this, it could be derived that recurrent, intense training will lead to both higher levels of those breakdown parameters and better future performance. Indeed, we found significant univariable associations between future performance and baseline ALAT (ρ = 0.526, p < .01) and BUN (ρ = 0.476, p < .05). Lippi et al. reported a significant univariable correlation between urea and running times in a half-marathon (r = -0.408, p = .007), but not for ALAT (r = -0.120, p = .442).[19] However, elevated activities of ALAT were observed by Petterson et al. for up to seven days post exercise.[42]

#### Total cholesterol

In our cohort, lower cholesterol was linked to better future performance (ρ = -0.460, p < .05). The lipid lowering capacity of distance running is well known in literature.[13] For marathoners, Williams described an intensity-dependent effect: male marathoners with longest usual weekly runs of ≥15 km presented with 34% lower odds of requiring anti-lipidemic treatment when compared to men running <5 km per week.[30] Kobayashi et al. registered a decrease of total cholesterol levels starting two days after a marathon run and lasting a week.[31] If all this is taken together, lower total cholesterol levels again might be an indicator of training volume.

#### Folic acid

In univariable analysis, folic acid levels were inversely related to future performance (ρ = 0.521, p < .01). Interestingly, Herrmann et al. registered a considerably high proportion of individuals with low pre-run folate levels among recreational athletes. Unfortunately, they did not report corresponding running times. As a possible mechanism, they suggested increased folate consumption during regular endurance exercise.[15] Two decades earlier, Matter et al. reported the same findings for female marathoners. According to them, substitution of folate did not affect performance.[14] Hence low circulating folate might well be a mere symptom of intensified training.

#### Myeloperoxidase

Myeloperoxidase is the main effector molecule of neutrophil antimicrobial response. Although its site of action is primarily the phagosome, myeloperoxidase can be released into the blood stream or the intercellular space as well, e.g. in the course of system inflammation,[35] as occurs during intensive endurance training. Suzuki et al. reported increased MPO levels immediately after marathon running. The resulting oxidative stress might be attenuated by simultaneously up-regulated anti-inflammatory cytokines.[16] A comparable increase of MPO during a marathon was also shown by Henrotin et al. However, the authors could not find any relationship between MPO levels and performance.[17] Interestingly, Hessel et al. reported that lipid peroxides remained increased for at least one week after a marathon.[18] Since MPO is considered a major catalyst for the initiation of lipid peroxidation,[43] their findings can be interpreted as being in line with our results.

#### The interplay between blood markers and physical performance

It is well established that physical exercise must be seen as a pre-analytical factor.[12] This means that exercise alters a broad variety of metabolic processes which are then reflected by specific alterations in blood markers. As a consequence, one’s physical habits must be taken into account when interpreting blood test results.[44]

During exhaustive exercise, tissue damage leads to the release of organ-specific markers into the bloodstream, which in turn causes an inflammatory response.[45] Moreover, reactive oxygen species (ROS) are produced and regulate muscle contraction and fatigue.[46] As a compensatory response, recurrent physical activity leads to elevated resistance against the oxidative stress caused by an increase in ROS.[46] At the same time, the consumption of blood lipids increases as a result to enhanced lipoprotein lipase activity [47] and lipoprotein particle composition [48]. Likewise, the increment in metabolic turnover might consume a higher amount of folate, which is a common cofactor in various metabolic reactions.[15]

Of note, our data does not provide evidence for causal relationships between the assessed blood markers and future fitness decrement. It is more likely that both, fitness decrement and changes in blood test levels depend at least partly on the same multiple factors, as e.g. on training volume, nutrition and recovery time [12]. As a result, different training efficacies might 1) influence the degree to which the exercise induced blood changes occur and 2) simultaneously determine subsequent fitness states. This enables the usage of the affected biochemical parameters as surrogates for training motivation, allowing for prediction of future running performance.

### Scope of applicability

To the best of our knowledge, this is the first study reporting a statistical model for predicting future performance changes using a set of blood parameters in a cohort of elderly marathon runners. Our results can be considered as highly valid for the cohort of elderly marathon runners, since the model’s goodness of fit was comparably high in both the model training and the test sample (p = 0.134). However, future investigations must examine whether the model can be translated to other disciplines and age cohorts.

As mentioned above, not many studies have been conducted investigating the predictability of future physical performance and only a handful concentrate on laboratory parameters. Knechtle et al. aimed to predict half-marathon race times by percent body fat and running speed during training, both assessed three months prior to competition. R^2^ were 0.42 for male and 0.68 for female runners.[9] In comparison, our model reached a Nagelkerke’s R^2^ = 0.789 within the model training sample. Regarding laboratory results, Lippi et al. reported correlations between mean platelet volumes and α-amylase levels which were assessed immediately before marathon running and running times. In univariate analysis, MPV correlated with running times by r = 0.450 (p = 0.002) and α-amylase by r = -0.598 (p<0.001). After controlling for anthropometric variables, p-values rose to 0.042 and 0.021 respectively.[6, 19] Unfortunately, the authors did not report any effect sizes for multivariable analysis which would allow comparison with our model. Hence it can only be stated that Lippi yielded univariate correlation coefficients comparable with our results. Bobbert et al. reported significant correlations between leptin assessed two days before a marathon and marathon times after adjusting for age and BMI (r = 0.412, p = 0.036).[20] A correlation coefficient of 0.412 corresponds to an R^2^ of 0.170, which is well below the R^2^ of our model.

It must be emphasized that our study did not aim to predict future marathon times, but general physical performance. However, there is good evidence that treadmill performance can be directly linked to running times. In this context, Till et al. estimated marathon running times by results from treadmill tests which took place two weeks prior to the marathon race.[49] In detail, the treadmilling protocol contained a 5-minute warm-up period at a 4% incline and a speed of 4km/h followed by stepwise progression every three minutes until voluntary exhaustion. Together with other parameters, treadmill times were further used to predict marathon times. Unlike sex, running hours per week, years of running and age, treadmill time was the only significant predictor (F = 8.19, p<0.01) of marathon performance time (adjusted R^2^ = 0.447).

Studies assessing long-term predictability of physical performance in marathoners are simply not available. After replication in different cohorts, our model might provide a powerful tool for sportsmen and coaches during the adaption of training methods.

### Limitations of the study

Of course, the present study comes with several limitations. In this regard, the overall sample size can be considered as quite moderate. However, the number of study participants compares very well to other studies published in the field: Lippi et al. included 43 amateur runners,[6] Heil et al. enrolled 21 cyclists,[8] Bobbert et. al. studied 36 athletes,[20] and Till et al. reported results from 59 marathoners.[49] Moreover, the sample size was obviously large enough to allow for statistically significant results, thus the sample could not be considered to be underpowered. Furthermore, the proportion of female participants is relatively low. Nevertheless, the composition of the cohort is representative for the overall population of elderly Austrian marathon runners: among marathoners above the age of 60 participating in the 2015 Vienna City Marathon, only 4.7% were female.

### Conclusion

Standard laboratory parameters could indeed be useful in monitoring training efficacy, as our findings suggest. It should be emphasized that our findings must not be interpreted in the sense that athletes should seek higher levels of muscle breakdown parameters or oxidative stress in order to enhance their future physical performance. Rather, the composition of the circulating analytes is more or less a mere symptom of recurring endurance training, which might lead to the desired capacity, and could thus be seen as a surrogate for persistent training motivation. Of course, further investigations on independent cohorts are necessary to assess the limits of our model’s applicability.
